# Natural Image Coding in V1: How Much Use Is Orientation Selectivity?

**DOI:** 10.1371/journal.pcbi.1000336

**Published:** 2009-04-03

**Authors:** Jan Eichhorn, Fabian Sinz, Matthias Bethge

**Affiliations:** Max Planck Institute for Biological Cybernetics, Tübingen, Germany; University College London, United Kingdom

## Abstract

Orientation selectivity is the most striking feature of simple cell coding in V1 that has been shown to emerge from the reduction of higher-order correlations in natural images in a large variety of statistical image models. The most parsimonious one among these models is linear Independent Component Analysis (ICA), whereas second-order decorrelation transformations such as Principal Component Analysis (PCA) do not yield oriented filters. Because of this finding, it has been suggested that the emergence of orientation selectivity may be explained by higher-order redundancy reduction. To assess the tenability of this hypothesis, it is an important empirical question how much more redundancy can be removed with ICA in comparison to PCA or other second-order decorrelation methods. Although some previous studies have concluded that the amount of higher-order correlation in natural images is generally insignificant, other studies reported an extra gain for ICA of more than 100%. A consistent conclusion about the role of higher-order correlations in natural images can be reached only by the development of reliable quantitative evaluation methods. Here, we present a very careful and comprehensive analysis using three evaluation criteria related to redundancy reduction: In addition to the multi-information and the average log-loss, we compute complete rate–distortion curves for ICA in comparison with PCA. Without exception, we find that the advantage of the ICA filters is small. At the same time, we show that a simple spherically symmetric distribution with only two parameters can fit the data significantly better than the probabilistic model underlying ICA. This finding suggests that, although the amount of higher-order correlation in natural images can in fact be significant, the feature of orientation selectivity does not yield a large contribution to redundancy reduction within the linear filter bank models of V1 simple cells.

## Author Summary

Since the Nobel Prize winning work of Hubel and Wiesel it has been known that orientation selectivity is an important feature of simple cells in the primary visual cortex. The standard description of this stage of visual processing is that of a linear filter bank where each neuron responds to an oriented edge at a certain location within the visual field. From a vision scientist's point of view, we would like to understand why an orientation selective filter bank provides a *useful* image representation. Several previous studies have shown that orientation selectivity arises when the individual filter shapes are optimized according to the statistics of natural images. Here, we investigate quantitatively how critical the feature of orientation selectivity is for this optimization. We find that there is a large range of non-oriented filter shapes that perform nearly as well as the optimal orientation selective filters. We conclude that the standard filter bank model is not suitable to reveal a strong link between orientation selectivity and the statistics of natural images. Thus, to understand the role of orientation selectivity in the primary visual cortex, we will have to develop more sophisticated, nonlinear models of natural images.

## Introduction

It is a long standing hypothesis that neural representations in sensory systems are adapted to the statistical regularities of the environment [1],[2]. Despite widespread agreement that neural processing in the early visual system must be influenced by the statistics of natural images, there are many different viewpoints on how to precisely formulate the computational goal the system is trying to achieve. At the same time, different goals might be achieved by the same optimization criterion or learning principle. Redundancy reduction [2], the most prominent example of such a principle, can be beneficial in various ways: it can help to maximize the information to be sent through a channel of limited capacity [3],[4], it can be used to learn the statistics of the input [5] or to facilitate pattern recognition [6].

Besides redundancy reduction, a variety of other interesting criteria such as *sparseness* [7],[8], *temporal coherence* [9], *predictive information* [10],[11] , or *bottom-up saliency* [12] have been formulated. An important commonality among all these ideas is the tight link to density estimation of the input signal.

At the level of primary visual cortex there is a large increase in the number of neurons. Hence, at this stage the idea of redundancy reduction cannot be motivated by a need for compression. However, the redundancy reduction principle is not limited to be useful for compression only. More generally, it can be interpreted as a special form of density estimation where the goal is to model the statistics of the input by finding a mapping which transforms the data into a representation with statistically independent coefficients [5]. In statistics, this idea is known as projection pursuit density estimation [13] where density estimation is carried out by optimizing over a set of possible transformations in order to match the statistics of the transformed signal as good as possible to a pre-specified target distribution. Once the distribution has been matched, applying the inverse transformation effectively yields a density model for the original data. From a neurobiological point of view, we may think of the neural response properties as an implementation of such transformations. Accordingly, we here think of redundancy reduction mainly in terms of projection pursuit density estimation.

A crucial aspect of this kind of approach is the class of transformations over which to optimize. From a statistician's point of view it is important to choose a regularized function space in order to avoid overfitting. On the other hand, if the class of possible transformations is too restricted, it may be impossible to find a good match to the target distribution. From a visual neuroscientist's point of view, the choice of transformations should be related to the class of possible computations in the early visual system. Here we assume the simplest case of linear transformations, optionally followed by a pointwise nonlinearity.

Intriguingly, a number of response properties of visual neurons have been reproduced by optimizing over the class of linear transformations on natural images for redundancy reduction (for a review see [12],[14]). For instance, Buchsbaum and Gottschalk as well as Ruderman et al. revealed a link between the second-order statistics of color images and opponent color coding of retinal ganglion cells by demonstrating that decorrelating natural images in the trichromatic color space with Principal Component Analysis (PCA) yields the luminance, the red-green, and the blue-yellow channel [15],[16]. Atick and Redlich derived the center-surround receptive fields by optimizing a symmetric decorrelation transformation [17]. Later, also spatio-temporal correlations in natural images or sequences of natural images were linked to the receptive field properties in the retina and the lateral geniculate nucleus (LGN) [18]–[20].

On the way from LGN to primary visual cortex, orientation selectivity emerges as a striking new receptive field property. A number of researchers (e.g., [21],[22]) have used the covariance properties of natural images to derive linear basis functions that exhibit similar properties. Decorrelation alone, however, was not sufficient to achieve this goal. Rather, additional constraints were necessary, such as spatial locality or symmetry.

It was not until the reduction of higher-order correlations were taken into account that the derivation of localized and oriented band-pass filters—resembling orientation selective receptive fields in V1— was achieved without the necessity to assume any further constraints. Those filters were derived with Independent Component Analysis (ICA), a generalization of Principal Component Analysis (PCA), which aims at reducing higher-order correlations as well [8],[23].

This finding suggests that within the linear filter model, orientation selectivity can serve as a further mechanism for redundancy reduction. The tenability of this hypothesis can be tested by measuring how large the advantage of orientation selective filters is over non-oriented filter shapes. The importance of such a *quantitative* assessment has first been pointed out by Li and Atick [22] and are the main focus of several publications [12], [22], [24]–[29]. Generally speaking, two different approaches have been taken in the past: In the first approach, nonparametric methods such as histograms or nearest neighbor statistics have been used with the goal to estimate the total redundancy of natural images [22],[27],[29]. While this approach seeks to answer the more difficult question how large the total redundancy of natural images is, the second approach compares the importance of orientation selectivity for redundancy reduction only within the class of models that are commonly used to describe V1 simple cell responses [24]–[26],[28].

Using histogram estimators, Zhaoping and coworkers [22],[27] argued that the contribution of higher-order correlations to the redundancy of natural images is five times smaller than the amount of second-order correlations. They concluded that this amount is so small that higher-order redundancy minimization is unlikely to be the main principle in shaping the cortical receptive fields.

Two objections may be raised against this conclusion: First, it is not clear how generally valid the result of [27] is. The study relies on the assumption that higher-order dependencies at distances beyond three pixels are negligible. More recent work based on nearest neighbor methods [29], however, finds a substantially larger amount of higher-order correlations when taking dependencies over longer distances into account. Secondly, even if the contribution of higher-order correlation was only 20% of the amount of second-order correlations, this contribution is not necessarily negligible. Several previous studies report that the redundancy reduction achieved with ICA for gray level images is at the same level at about 20% [24]–[26]. Taken together these two findings suggest that orientation selective ICA filters can account for virtually all higher-order correlations in natural images. If this was true, it would strongly support the idea that redundancy reduction could be the main principle in shaping the cortical receptive fields.

In general, however, density estimation in high dimensions is a hard problem and the results reported in the literature do not fit into a consistent view. Therefore, the crucial challenge is to control for all technical issues in order to allow for safe conclusions about the effect of orientation selectivity on redundancy reduction. Here, we address many such issues that have not been addressed before. In our study, we take the second approach and focus on “linear redundancy reduction”—the removal of statistical dependencies that can be achieved by linear filtering. While most studies have been carried out for gray level images the two studies on color images find the advantage of ICA over PCA to be many times larger for color images than for gray level images with an improvement of more than 100% [25],[26]. Since it is not clear how to explain the large difference between color and gray value images, we reinvestigate the comparison between the orientation selective ICA filters and the PCA filters for color images using the same data set as in [25],[26].

Our goal is to establish a reliable reference against which more sophisticated image models can be compared to in the future. We elaborate on our own previous work [28] by optimizing the ICA algorithm for the multi-information estimators used in the comparison. Additionally, we now test the advantage of the resulting orientation selective ICA filters comprehensively with three different types of analyses that are related to the notion of redundancy reduction, density estimation, and coding efficiency: (A) multi-information reduction, (B) average log-likelihood, and (C) rate-distortion curves.

Our results show that orientation selective ICA filters do not excel in any of these measures: We find that the gain of ICA in redundancy reduction over a random decorrelation method is only about 3% for color and gray-value images. In terms of rate-distortion curves, ICA performs even worse than PCA. Furthermore, we demonstrate that a simple spherically symmetric model with only two parameters fits the filter responses significantly better than a model that assumes marginal independence . Since in this model the specific shape of the filters is ignored, we conclude that it is unlikely that orientation selectivity plays a critical role for redundancy reduction even if the class of transformations is extended to include contrast gain control mechanisms [30],[31]. While many of the previous studies do not provide enough detail in order to explain their different outcomes, we provide our code and the dataset online (http://www.kyb.tuebingen.mpg.de/bethge/code/QICA/) in order to ensure the reproducibility and verifiability of our results.

## Materials and Methods

An important difficulty in setting up a quantitative comparison originates from the fact that it bears several issues that may be critical for the results. In particular, choices have to be made regarding the *evaluation criteria*, the *image data*, the *estimation methods*, which *linear transformations* to include in the comparison, and which *particular implementation of ICA* to use. The significance of the outcome of the comparison will depend on how careful these choices have been made. The most relevant issues will be addressed in the following.

### Notation and Nomenclature

For both, color and gray-value data, we write 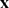 to refer to single vectors which contain the raw pixel intensities. Vectors are indicated by bold font while the same letter in normal font with a subindex denotes one of its components. Vectors without subindices usually denote random variables, while subindices indicate specific examples. In some cases it is convenient to define the corresponding data matrix 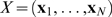 which holds single images patches in its columns. The letter 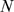 denotes the number of examples in the dataset, while 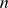 is used for the dimension of a single data point.

Transformations are denoted by 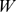, oftentimes with a subindex to distinguish different types. The result of a transformation to either a vector 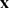 or a data matrix 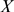 will be written as 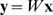 or 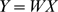, respectively.

Probability densities are denoted with the letters 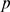 and 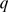, sometimes with a subindex to indicate differences between distributions whenever it seems necessary for clarity. In general, we use the hat symbol to distinguish between true entities and their empirical estimates. For instance, 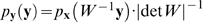 is the true probability density of 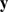 after applying a fixed transformation 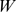, while 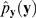 refers to the corresponding empirical estimate.

A distribution 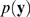 is called *factorial*, or *marginally independent*, if it can be written as a product of its marginals, i.e., 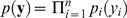 where 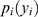 is obtained by integrating 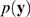 over all components but 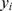.

Finally, the expectation over some entity 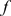 with respect to 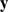 is written as 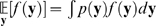. Sometimes, we use the density instead of the random variable in the subindex to indicate the distribution, over which the expectation is taken. If there is no risk for confusion we drop the subindex. Just as above, the empirical expectation is marked with a hat symbol, i.e., 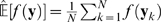.

### How to Compare Early Vision Models?

A principal complicacy in low-level vision is the lack of a clearly defined task. Therefore, it is difficult to compare different image representations as it is not obvious *a priori* what measure should be used.

#### Multi-information

The first measure we consider is the *multi-information* [32], which is the original objective function that is minimized by ICA over the choice of filters 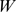. The multi-information assesses the total amount of statistical dependencies between the components 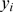 of a filtered patch 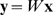:
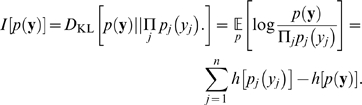
(1)The terms 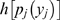 and 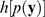 denote the marginal and the joint entropies of the true distribution, respectively. The *Kullback-Leibler-Divergence* or *Relative Entropy*

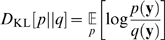
is an information theoretic dissimilarity measure between two distributions 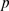 and 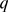 [33]. It is always non-negative and zero if and only if 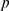 equals 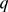. If the redundancy reduction hypothesis is taken literally, the multi-information is the right measure to minimize, since it measures how close to factorial the true distribution of the image patches in the representation 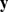 really is.

The application of linear ICA algorithms to ensembles of natural images reliably yields transformations consisting of localized and oriented bandpass filters similar to the receptive fields of neurons in V1. It is less clear, however, whether these filter properties also critical to the minimization of the multi-information? In order to assess the tenability of the idea that a V1 simple cell is adjusted to the purpose of redundancy reduction, it is important to know whether such a tuning can—*in principle*—result in a large reduction of the multi-information. One way to address this question is to measure *how much* more the multi-information is actually reduced by the ICA filters in comparison to others such as PCA filters. This approach has been taken in [28].

One problem with estimating multi-information is that it involves the joint entropy 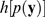 of the true distribution which is generally hard to estimate. In certain cases, however, the problem can be bypassed by evaluating the difference in the multi-information between two representations 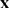 and 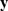. In particular, if 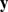 is related to 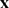 by the linear transformation 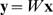 it follows from definition (1) and the transformation theorem for probability densities
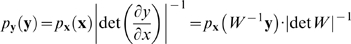
that difference in multi-information can be expressed as
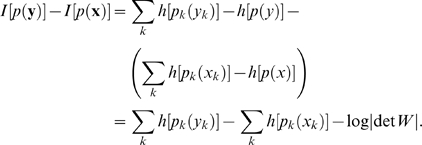
For convenience, we chose a volume-conserving gauge [28] where all linear decorrelation transforms are of determinant one, and hence 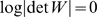. This means that differences in multi-information are equal to differences of marginal entropies which can be estimated robustly. Thus, our empirical estimates of the multi-information differences are given by:
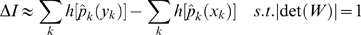
(2)For estimating the entropy of the univariate marginal distributions, we employ the OPT estimator introduced in [28] which uses the exponential power family to fit the marginal distributions by OPTimizing over the shape parameter. This estimator has been shown to give highly reliable results for natural images. In particular, it is much more robust than entropy estimators based on the sample kurtosis which easily overestimate the multi-information.

#### Average log loss (ALL)

As mentioned earlier, redundancy reduction can be interpreted as a special form of density estimation where the goal is to find a mapping which transforms the data into a representation with statistically independent coefficients. This means that any given transformation specifies a density model over the data. Our second measure, the average log-loss (ALL), evaluates the agreement of this density model with the actual distribution of the data:
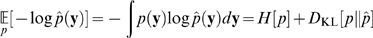
(3)The average log-loss is a principled measure quantifying how different the model density 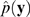 is from the true density 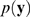 [34]. Since the KL-divergence is positive and zero if and only if 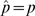 the ALL is minimal only if 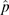 matches the true density. Furthermore, differences in the average log-loss correspond to differences in the coding cost (i.e., information rate) in the case of sufficiently fine quantization. For natural images, different image representations have been compared with respect to this measure in [24]–[26].

For the estimation of the average log-loss, we compute the empirical average
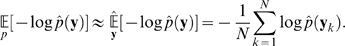
(4)This estimator is equivalent to the first method in Lewicki et al. [24],[35] apart from an extra term 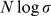 in their defining equation. This extra term is only necessary if one aims at relating the result to a discrete entropy obtained for a particular bin width 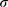.

While the empirical average in Eq. 4 in principle can be prone to overfitting, we control for this risk by evaluating all estimates on an independent test set, whose data has not been used during the parameter fit. Furthermore, we compare the average log-loss to the parametric entropy estimates 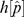 that we use in (A) for estimating the multi-information changes (see Eq. 2). The difference between both quantities has been named *differential log-likelihood* [36] and can be used to assess the goodness of fit of a model distribution:
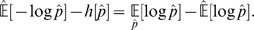



The shape of the parametric model is well matched to the actual distribution if the differential log-likelihood converges to zero with increasing number of data points.

#### Rate-distortion curves

Finally, we consider *efficient coding* or *minimum mean square error reconstruction* as a third objective. In contrast to the previous objectives, it is now assumed that there is some limitation of the amount of information that can be transmitted, and the goal is to maximize the amount of *relevant* information transmitted about the image. In the context of neural coding, the redundancy reduction hypothesis has oftentimes been motivated in terms of coding efficiency. In fact, instead of minimizing the multi-information one can equivalently ask for the linear transformation 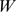 which maximizes the mutual information between its input 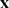 and its output 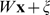 when additive noise 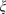 is added to the output [3],[37],[38]. It is important to note, however, that this minimalist approach of “information maximization” is ignorant with respect to how useful or *relevant* the information is that has been transmitted [14].

For natural images, the source signal 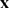 is a continuous random variable which requires infinitely many bits to be specified with unlimited precision. In reality, however, the precision is always limited so that only a finite amount of bits can be represented. Both, the multi-information and the average log-loss do not take into account the problem what information should be encoded and what information can be discarded. Therefore, it is interesting to compare the redundancy reduction of the linear transforms with respect to the relevant image information (while the irrelevant information can be discarded anyway). To this end, we here resort to the framework of linear transform coding as it has been developed in the field of image compression [39],[40], and which constitutes the theoretical foundation of the JPEG standard.

It is clear that at the level of V1 the number of neurons, encoding the retinal image, is substantially larger than the number of fibers in the optic nerve. Therefore, it is not the need for compression that makes rate distortion theory interesting at this stage. However, Barlow's redundancy reduction hypothesis must not be equated with compression. In more recent work, Barlow introduced the term ‘redundancy exploitation’ instead of ‘redundancy reduction’ in order to avoid this misunderstanding [41]. But also if we think in terms of density estimation rather than compression, it is still important to take into account that not all possible changes in the image pixels may be of equal importance for inferring the content of an image. Therefore, we here want to combine the notion of redundancy reduction with a measure for the quality with which the image can be reconstructed from the information that is preserved by the representation. Following Lewicki and coworkers (method 2 in [24],[35]) we will consider the mean squared error reconstruction that can be achieved at a certain quantization level of the transformed representation. This objective is in fact very much related to the task of image compression.

Clearly, we expect that the criteria for judging image compression algorithms may not provide a good proxy to an accurate judgement of what information is considered relevant in a biological vision system. In particular, the existence of selective attention suggests that different aspects of image information are transmitted at different times depending on the behavioral goals and circumstances [12]. That is, a biological organism can change the relevance criteria dynamically on demand while for still image compression algorithms it is rather necessary that this assessment is made once and forever in a fixed and static fashion.

These issues are outside the scope of this paper. Instead we follow the common path in the past to use the mean squared reconstruction error for the pixel intensities. This is the measure of choice for high-rate still image compression [42]. In particular, it is common to report on the performance of a code by determining its rate–distortion curve which specifies the required information rate for a given reconstruction error (and vice versa) [40]. Consequently, we will ask for a given information rate, how do the image representations compare with respect to the reconstruction error. As result, we will obtain a so-called rate–distortion curve which displays the average reconstruction error as a function of the information rate or vice versa. The second method used in [24],[35] is an estimate of a single point on this curve for a particular fixed value of the reconstruction error.

The estimation of the rate–distortion curve is clearly the most difficult task among the three criteria. The framework of transform coding [39], which is extensively used in still image compression, makes several simplifying assumptions that allow one to obtain a clear picture. The encoding task is divided into two steps: First, the image patches 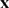 are linearly transformed into 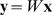. Then the coefficients 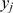 are quantized independently of each other. Using this framework, we can ask whether the use of an ICA image transformation leads to a smaller reconstruction error after coefficient quantization than PCA or any other transform.

As for quantizing the coefficients, we resort to the framework of variable rate entropy coding [43]. In particular, we apply uniform quantization, which is close to optimal for high-rate compression [39],[44]. For uniform quantization, it is only required to specify the bin width of the coefficients. There is also the possibility to use a different number of quantization levels for the different coefficients. The question of how to set these numbers is known as the ‘bit allocation problem’ because the amount of bits needed to encode one coefficient will depend monotonically on the number of quantization levels. The number of quantization levels can be adjusted in two different but equivalent ways: One possibility is to use a different bin width for each individual coefficient. Alternatively, it is also possible to use the same bin width for all coefficients and multiply all coefficients with an appropriate scale factor before quantization. The larger the variance of an individual coefficient, the more bits will be allocated to represent it.

Here, we will employ the latter approach, for which the bit allocation problem becomes an inherent part of the transformation: Any bit allocation scheme can be obtained via post-multiplication with a diagonal matrix. Thus, in contrast to the objective function of ICA, the rate–distortion criterion is not invariant against post-multiplication with a diagonal matrix. For ICA and PCA, we will determine the rate–distortion curve for both, normalized output variances (“white ICA” and “white PCA”) and normalized basis functions (“normalized ICA” and “orthonormal PCA”), respectively.

### Decorrelation Transforms

The particular shape of the ICA basis functions is obtained by minimization of the multi-information over all invertible linear transforms 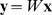. In contrast, the removal of second-order correlations alone generally does not yield localized, oriented, and bandpass image basis functions. ICA additionally removes higher-order correlations which are generated by linear mixing. In order to assess the importance of this type of higher-order correlations for redundancy reduction and coding efficiency we will compare ICA to other decorrelating image bases.

Let 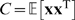 be the covariance matrix of the data and 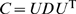 its eigen-decomposition. Then, any linear second-order decorrelation transform can be written as
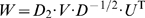
(5)where 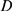 and 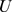 are defined as above, 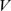 is an arbitrary orthogonal matrix and 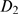 is an arbitrary diagonal matrix. It is easily verified that 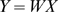 has diagonal covariance for all choices of 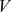 and 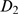, i.e., all second-order correlations vanish. This means that any particular choice of 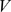 and 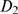 determines a specific decorrelation transform. Based on this observation we introduce a number of linear transformations for later reference. All matrices are square and are chosen to be of determinant 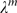, where 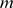 is the number of columns (or rows) of 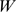 (i.e., 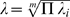 is the geometrical mean of the eigenvalues 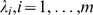).

#### Orthogonal principal component analysis (oPCA)

If the variances of the principle components (i.e., the diagonal elements of 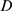) are all different, PCA is the only metric-preserving decorrelation transform and is heavily used in digital image coding. It corresponds to choosing 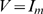 as the identity matrix and 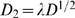, such that 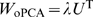.

#### White principal component analysis (wPCA)

Equalizing the output variances in the PCA representation sets the stage for the derivation of further decorrelation transforms different from PCA. In order to assess the effect of variance equalization for coding efficiency, we also include this “white PCA” representation into our analysis: Choose 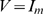 as for orthonormal PCA and then set 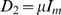 with 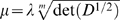 such that 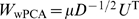.

#### Symmetric whitening (SYM)

Among the non-orthogonal decorrelation transforms, symmetric whitening stays as close to the input representation as possible (in Frobenius norm) [45]. In terms of early vision this may be seen as an implementation of a wiring length minimization principle. Remarkably, the basis functions of symmetric whitening resemble the center-surround shape of retinal ganglion cell receptive fields when applied to the pixel representation of natural images [17]. The symmetric whitening transform is obtained by setting 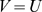 and 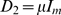 such that 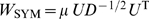.

#### Random whitening (RND)

As a baseline which neither exploits a special structure with respect to the input representation nor makes use of higher-order correlations we also consider a completely random transformation. To obtain a random orthogonal matrix we first draw a random matrix 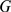 from a Gaussian matrix-variate distribution and then we set 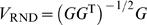. With 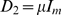 we obtain 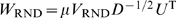.

#### White independent component analysis (wICA)

Finally, ICA is the transformation which has been suggested to explain the orientation selectivity of V1 simple cells [8],[23]. Set 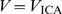 for which the multi-information 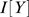 takes a minimum. With 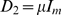 we obtain 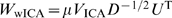.

#### Normalized independent component analysis (nICA)

Normalized independent component analysis (nICA) differs from white ICA (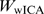) only by a different choice of the second diagonal matrix 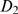. Instead of having equal variance in each coefficient, we now choose 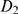 such that the corresponding basis vector of each coefficient has the same length in pixel space. It is easy to see that our first two criteria, the multi-information and the negative log-likelihood, are invariant under changes in 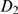. It makes a difference for the rate–distortion curves as in our setup the variance (or, more precisely, the standard deviation) determines the bit allocation. Practically, 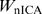 can be determined by using 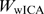 as follows: First, we compute the matrix inverse 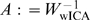 and determine the Euclidean norm 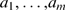 of the column vectors of 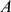. With 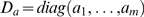, we then obtain 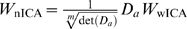.

### ICA Algorithm

If the true joint probability distribution is known, the minimization of the multi-information over all linear transformations can be formulated without any assumptions about the shape of the distribution. In practice, the multi-information has to be estimated from a finite amount of data which requires to make assumptions about the underlying density.

There are many different ICA algorithms which differ in the assumptions made and also in the optimization technique employed. The choice of the particular ICA algorithm used here was guided by a set of requirements that arise from the specific problem setting. Although a wide variety of ICA algorithms has been published, none of them fits exactly all of our requirements.

We would like to use an ICA algorithm, which gives the ICA image basis the best chance for the comparison with other image representations. For the comparison of the multi-information reduction, we are using the OPT estimator introduced in [28] which has been found to give the most reliable results. This estimator employs a parametric estimate of the coefficient distributions based on the exponential power family which is known to provide an excellent fit to the coefficient distributions of natural images [28],[46]. Our ICA algorithm should make the same assumptions about the data as we make for the final comparison of the multi-information reduction. Therefore, we are also using the exponential power family model for the marginal densities during the minimization of the multi-information. In addition, we want to have an ICA basis which is indistinguishable from the other image representations with respect to the second-order statistics. Therefore, we are using a pre-whitened ICA algorithm, whose search space is restricted to the subgroup of orthogonal matrices 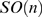. One of the most efficient ICA methods in the public domain specialized to pre-whitened ICA is FastICA [47]. We use this fixed-point algorithm as an initialization. Subsequently, the solution is further refined by performing a gradient ascent over the manifold of orthogonal matrices on the likelihood of the data, when each marginal is modelled by a the exponential power distribution as in the case of the OPT estimator.

In order to optimize the objective function over the subspace of orthogonal matrices, we adapted the algorithms for Stiefel manifolds proposed by Edelman et al. [48] to the simpler case of orthogonal groups and combined it with the line-search routine dbrent from [49] to achieve a rather straightforward gradient descent algorithm. For the initialization with FastICA, we use the Gaussian non-linearity, the symmetric approach and a tolerance level of 10^−5^.

### Spherically Symmetric Model

A well known result by Maxwell [50] states that the only factorial distribution invariant against arbitrary orthogonal transformations is the isotropic Gaussian distribution. Natural images exhibit marginals which are significantly more peaked than Gaussian. Nevertheless, their distribution does share the spherical symmetry with the Gaussian as already found by [51] for gabor filter pairs and lately exploited by [31] for nonlinear image representations. Therefore, it makes sense to compare the performance of the ICA model with a spherically symmetric model of the whitened data 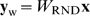. Note that any spherically symmetric model is still invariant under orthogonal transformations while only the Gaussian additionally exhibits marginal independence.

While the radial distribution of a Gaussian (i.e., the distribution over the lengths of the random vectors) is a 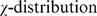, whose shape and scale parameter is determined by the number of dimensions and the variance, respectively, the spherical symmetric model may be seen as a generalization of the Gaussian, for which the radial distribution 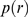 with 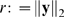 can be of arbitrary shape. The density of the spherically symmetric distribution (SSD) is defined as 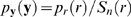, where 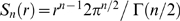 is the surface area of a sphere in 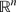 with radius 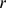. For simplicity we will model the radial distribution with a member of the Gamma family
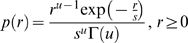
(6)with shape parameter 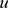 and scale parameter 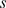, which can be easily matched to the mean and variance of the empirical distribution via 
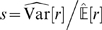
 and 
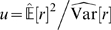
.

### Dataset

The difference in the performance between ICA and other linear transformations clearly depends on the data. For *gray-scale* images we observed in our previous study [28] that the difference in the multi-information between ICA and any other decorrelation transform is consistently smaller than 5%. In particular, we controlled for the use of different pictures and for the effect of different pre-processing steps.

Here, we resort to the dataset used in a previous study [25],[26], which among all previous studies reported the largest advantage of ICA compared to PCA. This *color* image dataset is based on the Bristol Hyperspectral Images Database [52] that contains multi-spectral recordings of natural scenes taken in the surroundings of Bristol, UK and in the greenhouses of Bristol Botanical Gardens. The authors of [26] kindly provided to us a pre-processed version of the image data where spectral radiance vectors were already converted into LMS values. During subsequent processing the reflectance standard was cut out and images were converted to log intensities [26].

All images come at a resolution of 256×256 pixels. From each image circa 5000 patches of size 7×7 pixels were drawn at random locations (circa 40000 patches in total). For chromatic images with three color channels (LMS) each patch is reshaped as a 7×7×3 = 147-dimensional vector. To estimate the contribution of color information, a comparison with monochromatic images was performed where gray-value intensities were computed as 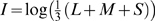 and exactly the same patches were used for analysis. In the latter case, the dimensionality of a data sample is thus reduced to 49 dimensions. All experiments are carried out over ten different training and test sets sampled independently from the original images.

Our motivation to chose 7×7 patches is to keep the same setting as in [26] for the sake of comparability. As this patch size is rather small, we performed the same analysis for patch sizes of 15×15 as well. All results in the paper refer to the case of 7×7 image patches. The results for 15×15 can be found in the supplementary material (Text S1).

The statistics of the average illumation in the image patches, the DC component, differs significantly from image to image. Therefore, we first separated the DC component from the patches before further transforming them. In order to leave the entropy of the data unaffected, we used an orthogonal transformation. The projector 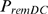 is computed such that the first (for each color channel) component of 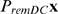 corresponds to the DC component(s) of that patch. One such a possible choice is the matrix
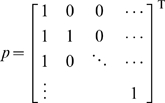
However, this is not an orthogonal transformation. Therefore, we decompose 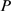 into 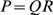 where 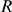 is upper triangular and 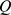 is an orthogonal transform. Since 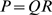, the first column of 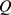 must be a multiple of the vector with all coefficients equal to one (due to the upper triangluarity of 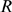). Therefore, the first component of 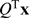 is a multiple of the DC component. Since 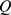 is an orthonomal transform, using all but the first row of 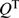 for 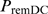 projects out the DC component. In the case of color images 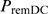 becomes a block-diagonal matrix with 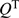 as diagonal elements for each channel.

By removing the DC component in that manner, all linear transformations are applied in 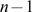 dimensions, if 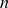 denotes the number of pixels in the original image patch. In this case the marginal entropy of the DC-components has to be included in the computation of the multi-information in order to ensure a valid comparison with the original pixel basis. We use the same estimators as in [28] to estimate the marginal entropy of DC-component.

## Results

### Filter Shapes

**Figure 1:**
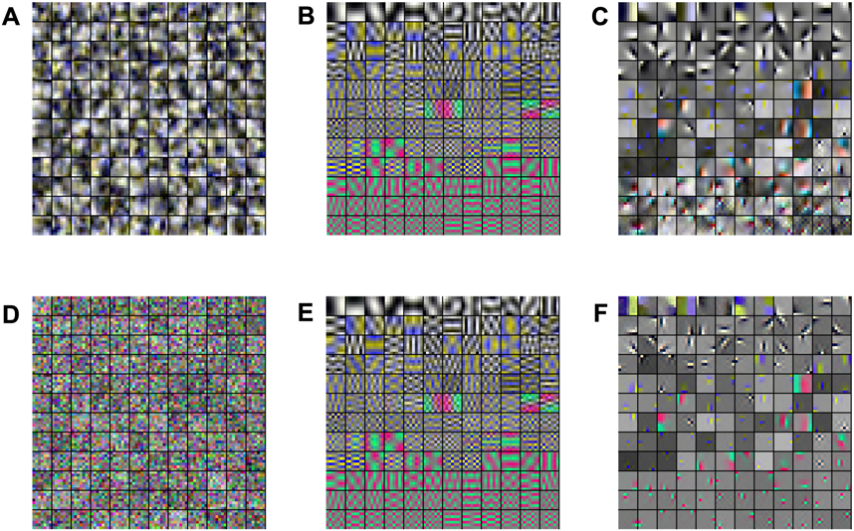
Examples for Receptive Fields of Various Image Transforms. Basis functions of a random decorrelation transform (RND), principal component analysis (PCA) and independent component analysis (ICA) in pixel space (A–C) and whitened space (E–F). The image representation in whitened space is obtained by left multiplication with the matrix square root of the inverse covariance matrix 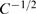.

As in previous studies [8],[23] the filters derived with ICA exhibited orientation selective tuning properties similar to those observed for V1 simple cells (see Figure 1). For illustration, we also show the basis functions learned with PCA and RND in Figure 1. The basis functions 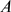 are obtained by inverting the filter matrix 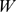 (including the DC component). The result is displayed in the upper panel (Figure 1A–C). Following common practice, we also visualize the basis functions after symmetric whitening (Figure 1D–F).

The basis functions of both PCA and ICA exhibit color opponent coding but the basis functions of ICA are additionally localized and orientation selective. The basis functions of the random decorrelation transform does not exhibit any regular structure besides the fact that they are bandpass. The following quantitative comparisons will show, however, that the distinct shape of the ICA basis functions does not yield a clear advantage for redundancy reduction and coding efficiency.

### Multi-Information

**Figure 2:**
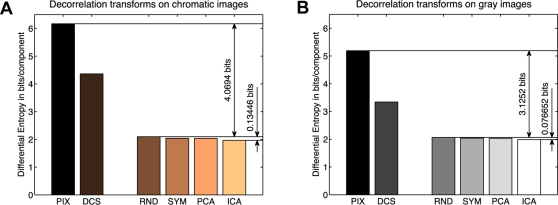
Multi-Information Reduction per Dimension. Average differential entropy 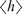 for the pixel basis (PIX), after separation of the DC component (DCS), and after application of the different decorrelation transforms. The difference between PIX and RND corresponds to the redundancy reduction that is achieved with a random second-order decorrelation transform. The small difference between RND and ICA is the maximal amount of higher-order redundancy reduction that can be achieved by ICA. Diagram (A) shows the results for chromatic images and diagram (B) for gray value images. For both types of images, only a marginal amount can be accounted to the reduction of higher order dependencies.

The multi-information is the original objective function that is minimized by ICA over all possible linear decorrelation transforms. Figure 2 shows the reduction in multi-information achieved with different decorrelation transforms including ICA for chromatic and gray value images, respectively. For each representation, the results are reported in bits per component, i.e., as marginal entropies averaged over all dimensions:
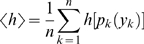
(7)


Table 1 shows the corresponding values for the transformations RND, SYM, PCA and ICA. For both chromatic images and gray-value intensities, the lowest and highest reduction is achieved with RND or ICA, respectively. However, the additional gain in the multi-information reduction achieved with ICA on top of RND constitutes only 3.20% for chromatic images and 2.39% for achromatic in comparison with the total reduction relative to the pixel basis (PIX). This means that only a small fraction of redundancy reduction can actually be accounted to the removal of higher-order redundancies with ICA.

**Table 1:** Comparision of the Multi-Information Reduction for Chromatic and Achromatic Images.

Absolute Difference			Relative Difference		
	Color	Gray		Color	Gray
RND-PIX	−4.0694±0.0043	−3.1252±0.0043			
SYM-RND	−0.0593±0.0004	−0.0259±0.0006	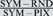	1.44±0.01	0.82±0.02
PCA-RND	−0.0627±0.0008	−0.0353±0.0011	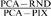	1.52±0.02	1.12±0.03
ICA-RND	−0.1345±0.0008	−0.0767±0.0008	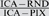	3.20±0.02	2.39±0.02

Differences in the multi-information reduction between various decorrelation transforms (SYM, PCA, ICA) relative to a random decorrelation transform (RND) compared to the multi-information reduction achieved with the random decorrelation transform relative to the original pixel basis (RND-PIX). The *absolute* multi-information reduction is given in bits/component on the left hand side. The right hand side shows how much more the special decorrelation transforms SYM, PCA and ICA can reduce the multi-information *relative* to the random (RND) one.

**Figure 3:**
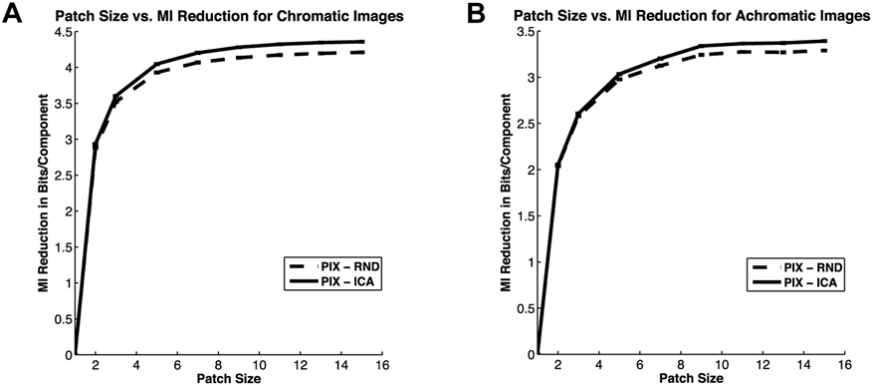
Redundancy Reduction as a Function of Patch Size. The graph shows the multi-information reduction achieved by the transformations RND and ICA for chromatic (A) and achromatic images (B). The gain quickly saturates with increasing patch size such that its value for 7×7 image patches is already at about 90% of its asymptote. This demonstrates that the advantage of ICA over other transformations does not increase with increasing patch size.

One may argue that the relatively small patch size of 7×7 pixel may be responsible for the small advantage of ICA as all decorrelation functions already getting the benefit of localization. In order to address the question how the patch size affects the linear redundancy reduction, we repeated the same analysis on a whole range of different patch sizes. Figure 3 shows the multi-information reduction with respect to the pixel representation (PIX) achieved by the transformations RND and ICA. The achievable reduction quickly saturates with increasing patch size such that its value for 7×7 image patches is already at about 90% of its asymptote. In particular, one can see that the relative advantage of ICA over other transformations is still small (∼3%) also for large patch sizes. All Tables and Figures for patch size 15×15 can be found in the additional material (Text S1).

### Average Log-Loss

Since redundancy reduction can also be interpreted as a special form of density estimation we also look at the average log-loss which quantifies how well the underlying density model of the different transformations is matched to the statistics of the data. Table 2 shows the average log-loss (ALL) and Table 3 the differential log-likelihood (DLL) in bits per component. For the average log-loss, ICA achieved an ALL of 1.78 bits per component for chromatic images and 1.85 bits per component for achromatic images. Compared to the ALL in the RND representation of 1.9 bits and 1.94 bits, respectively, the gain achieved by ICA is again small. Additionally, the ALL values were very close to the differential entropies, resulting in small DLL values. This confirms that the exponential power distribution fits the shape of the individual marginal coefficient distributions well. Therefore, we can safely conclude that the advantage of ICA is small not only in terms of redundancy reduction as measured by the multi-information, but also in the sense of density estimation.

**Table 2:** Average Log-Loss (ALL) for Chromatic and Achromatic Images.

	Color	Gray
	ALL	ALL
RND	1.9486±0.0035	1.9414±0.0044
SYM-RND	−0.0881±0.0004	−0.0402±0.0005
PCA-RND	−0.0751±0.0009	−0.0391±0.0011
ICA-RND	−0.1637±0.0007	−0.0880±0.0007
SSD-RND	−0.2761±0.0025	−0.2868±0.0032

The first row shows the average log-loss (ALL, in bits/component) of the density model determined by the linear transformation RND. The value was obtained by averaging over 10 separately sampled training and test sets of size 40.000 and 50.000, respectively. The following rows show the difference of the ALL of the models SYM, PCA, ICA and of the spherically symmetric density (SSD) to the ALL of the RND model. The smaller average log-loss of the SSD model compared to the ICA model fundamentally contradicts the assumptions underlying the ICA model.

**Table 3:** Differential Log-Likelihood (DLL) for Chromatic and Achromatic Images.

	Color		Gray	
	DLL	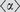	DLL	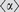
RND	−0.0113±0.0007	1.0413±0.0026	−0.0057±0.0006	1.0132±0.0046
SYM	−0.0388±0.0009	0.8961±0.0021	−0.0195±0.0009	0.9486±0.0040
PCA	−0.0224±0.0007	0.9145±0.0024	−0.0087±0.0007	0.9425±0.0025
ICA	−0.0378±0.0009	0.7687±0.0017	−0.0154±0.0011	0.8434±0.0025

The small DLL values suggest, that the exponential power distribution fits the shape of the individual coefficient distributions well. In addition, we also report the average exponent 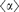 of the exponential power family fit to the individual coefficient distributions (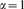 corresponds to a Laplacian shape).

#### Comparison to a Spherical Symmetric Model

The fact that ICA fits the distribution of natural images only marginally better than a random decorrelation transform implies that the generative model underlying ICA does not apply to natural images. In order to assess the importance of the actual filter shape, we fitted a spherically symmetric model to the filter responses. The likelihood of such a model is invariant under post-multiplication of an orthogonal matrix, i.e., the actual shape of the filter. Therefore, a good fit of such a model provides strong evidence against a critical role of certain filter shapes.

As shown in Table 2, the ALL of the SSD model is 1.67 bits per component for chromatic images and 1.65 bits per component for achromatic images. This is significantly smaller than the ALL of ICA indicating that it fits the distribution of natural images much better than ICA does. This result is particularly striking if one compares the number of parameters fitted in the ICA model compared to the SSD case: After whitening, the optimization in ICA is done over the manifold of orthogonal matrices which has 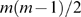 free parameters (where 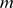 denotes the number of dimensions without the DC components). The additional optimization of the shape parameters for the exponential power family fitted to each individual component adds another 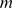 parameters. For the case of 7×7 color image patches we thus have 
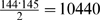
 parameters. In stark contrast, there are only two free parameters in the SSD model with a radial Gamma distribution, the shape parameter 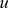 and the scale parameter 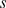. Nevertheless, for chromatic images the gain of the SSD model relative to random whitening is almost twice as large as that of ICA and even three and a half times as large for achromatic images.

Since the SSD model is completely independent of the choice of the orthogonal transformation after whitening, its superior performance compared with ICA provides a very strong argument against the hypothesis that orientation selectivity plays a critical role for redundancy reduction. In addition, it is also corroborates earlier arguments that has been given to show that the statistics of natural images does not conform to the generative model underlying ICA [51],[53].

**Figure 4:**
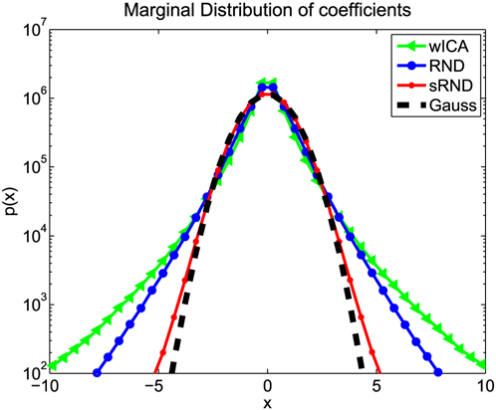
The Distribution of Natural Images does not Conform with the Generative Model of ICA. In order to test for statistical dependencies among the coefficients 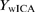 of whithened ICA for single data samples, the coefficients were shuffled among the data points along each dimension. Subsequently, we transform the resulting data matrix 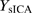 into 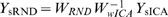. This corresponds to a change of basis from the ICA to the random decorrelation basis (RND). The plot shows the log-histogram over the coefficients over all dimensions. If the assumptions underlying ICA were correct, there would be no difference between the histogram of 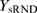 and 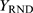.

Besides the better fit of the data by the SSD model, there is also a more direct way of demonstrating the dependencies of the ICA coefficients: If 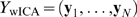 is data in the wICA representation, then the independence assumption of ICA can be simulated by applying independent random permutations to the rows of 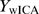. Certainly, such a shuffling procedure does not alter the histograms of the individual coefficients but it is suited to destroy potential statistical dependencies among the coefficients. Subsequently, we can transform the shuffled data 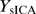 back to the RND basis 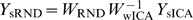. If the ICA coefficients were independent, the shuffling procedure would not alter the joint statistics, and hence, one should find no difference in the multi-information between 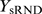 and 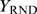. But infact, we observe a large discrepancy between the two (Figure 4). The distributions of the sRND coefficients were very close to Gaussians and the average marginal entropy of sRND yielded 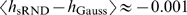 bits in contrast to 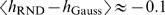 bits. In other words, the finding that for natural images the marginals of a random decorrelation transform have Laplacian shape (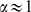) stands in clear contradiction to the generative model underlying ICA. If the ICA model was valid, one would expect that the sum over the ICA coefficients would yield Gaussian marginals due to the central limit theorem. In conclusion, we have very strong evidence that the ICA coefficients are not independent in case of natural images.

### Rate-Distortion Curves

There are different ways to account for the limited precision that is imposed by neural noise and firing rate limitations. As mentioned above the advantage with respect to a plain information maximization criterion can equivalently be measured by the multi-information criterion considered above [37],[54]. In order to additionally account for the question which representation optimally encodes the *relevant* image information, we also present rate distortion curves which show the minimal reconstruction error as a function of the information rate.

**Figure 5:**
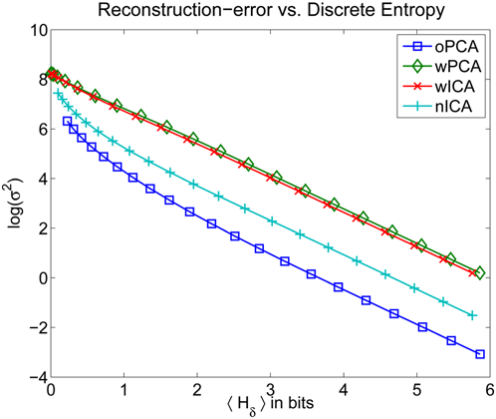
Rate-distortion Curves. Rate-distortion curve for PCA and ICA when equalizing the output variances (wPCA and wICA) and when equalizing the norm of the corresponding image bases in pixel space (oPCA and nICA). The plot shows the discrete entropy 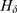 in bits (averaged over all dimensions) against the log of the squared reconstruction error 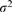. oPCA outperforms all other transforms in terms of the rate-distortion trade-off. wPCA in turn performes worst and remarkably similar to wICA. Since wPCA and wICA differ only by an orthogonal transformation, both representations are bound to the same metric. oPCA is the only transformation which has the same metric as the pixel representation according to which the reconstruction error is determined. By normalizing the length of the ICA basis vectors in the pixel space, the metric of nICA becomes more similar to the pixel basis and the performance with respect to the rate-distortion trade-off can be seen to improve considerably.

We compare the rate–distortion curves of wICA, nICA, wPCA and oPCA (see Figure 5). Despite the fact that ICA is optimal in terms of redundancy reduction (see Table 2), oPCA performs optimal with respect to the rate-distortion trade-off. wPCA in turn performes worst and remarkably similar to wICA. Since wPCA and wICA differ only by an orthogonal transformation, both representations are bound to the same metric. oPCA is the only transformation which has the same metric as the pixel representation according to which the reconstruction error is determined. By normalizing the length of the ICA basis vectors in the pixel space, the metric of nICA becomes more similar to the pixel basis and the performance with respect to the rate-distortion trade-off improved considerably. Nevertheless, for a fixed reconstrucion error the discrete entropy after quantization in the oPCA basis is up to 1 bit/component *smaller* than for the corresponding nICA-basis.

In order to understand this result more precisely, we analyzed how the quantization of the coefficients affects the two variables of the rate–distortion function, *discrete entropy* and *reconstruction error*.

**Figure 6:**
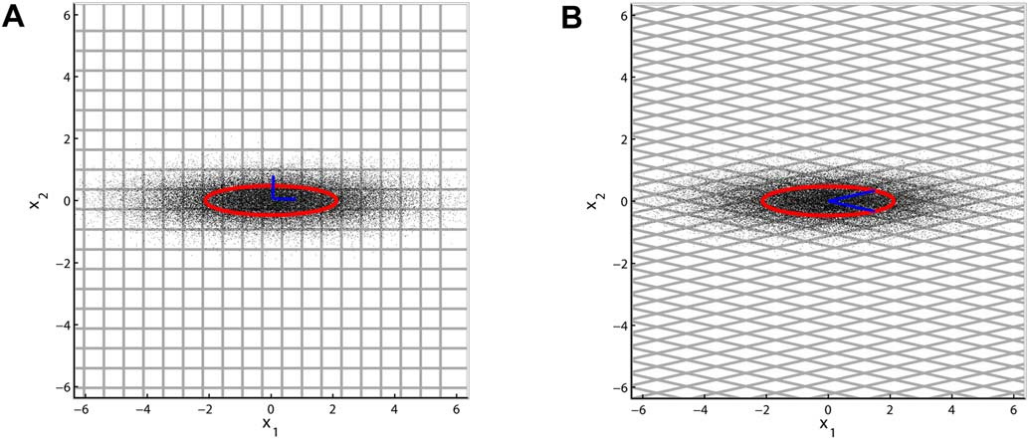
The Partition Cell Shape is Crucial for the Quantization Error. The quality of a source code depends on both the shapes of the partition cells and on how the sizes of the cells vary with respect to the source density. When the cells are small (i.e., the entropy rate is high), then, the quality mainly depends on having cell shapes that minimize the average distance to the center of the cell. For a given volume, a body in Euclidean space that minimizes the average distance to the center is a sphere. The best packings (including the hexagonal case) cannot be achieved with linear transform codes. Transform codes can only produce partitions into parallelepipeds, as shown here for two dimensions. The best parallelepipeds are cubes which are only obtained in the case of orthogonal transformations. Therefore PCA yields the (close to) optimal trade-off between minimizing the redundancy *and* the distortion as it is the only *orthogonal* decorrelation transform (see [39] for more details). The figure shows 50.000 samples from a bivariate Gaussian random variable. Plot (A) depicts a uniform binning (bin width 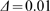, only some bin borders are shown) induced by the only orthogonal basis for which the coefficients 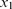 and 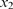 are decorrelated. Plot (B) shows uniform binning in a decorrelated, but not orthogonal basis (indicated by the blue lines). Both cases have been chosen such that the multi-information between the coefficients is identical and the same entropy rate was used to encode the signal. However, due to the shape of the bins in plot (B) the total quadratic error increases from 0.4169 to 0.9866. The code for this example can be also downloaded from http://www.kyb.tuebingen.mpg.de/bethge/code/QICA/.

Figure 6 shows an illustrative example in order to make the following analysis more intuitive. The example demonstrates that the quality of a transform code not only depends on the redundancy of the coefficients but also on the shape of the partition cells induced by the quantization. In particular, when the cells are small (i.e., the entropy rate is high), then the reconstruction error mainly depends on having cell shapes that minimize the average distance to the center of the cell. Linear transform codes can only produce partitions into parallelepipeds (Figure 6B). The best parallelepipeds are cubes (Figure 6A). This is why PCA yields the (close to) optimal trade-off between minimizing the redundancy *and* the distortion, as it is the only orthogonal transform that yields uncorrelated coefficients. For a more comprehensive introduction to transform coding we refer the reader to the excellent review by Goyal [39].

#### Discrete entropy

**Figure 7:**
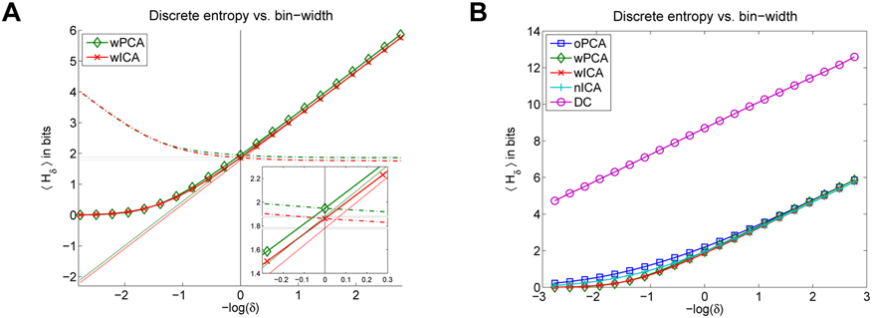
Discrete vs. Differential Entropy. (A) Relationship between discrete and differential entropy. Discrete entropy 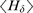 averaged over all channels as a function of the negative log bin width. The straight lines constitute the linear approximation to the asymptotic branch of the function. Their interception with the y-axis are visualized by the gray shaded, horizontal lines. The dashed lines represent 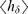 which converge to the gray shaded lines for 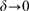. (B) There are only small differences in the average discrete entropy for oPCA, wPCA, wICA, nICA as a function of the negative log bin width. Since the discrete entropy of the DC component is the same for all transforms, it is not included in that average but plotted separately instead.

Given a uniform binning of width 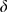 the discrete entropy 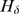 of a probability density 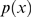 is defined as
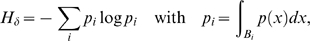
(8)where 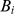 denotes the interval defined by the 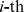 bin. For small bin-sizes 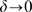, there is a close relationship between *discrete* and *differential* entropy: Because of the mean value theorem we can approximate 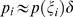 with 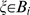, and hence
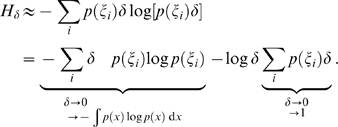
Thus, we have the relationship 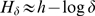 for sufficiently small 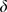 (i.e., high-rate quantization). In other words, 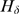 asymptotically grows linearly with 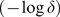. Therefore, we can fit a linear function to the asymptotic branch of the function 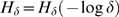 which is plotted in Figure 7A (more precisely we are plotting the average over all dimensions). If we take the ordinate intercept of the linear approximation, we obtain a nonparametric estimate of the differential entropy which can be compared to the entropy estimates reported above (Those estimates were determined with the OPT estimator). Equivalently, one can consider the function 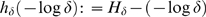 which gives a better visualization of the error of the linear approximation (Figure 7, left, dashed line). For 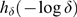 the differential entropy is obtained in the limit 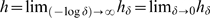.

This analysis shows that differences in differential entropy in fact translate into differences in discrete entropy after uniform quantization with sufficiently small bins. Accordingly, the minimization of the multi-information as proposed by the redundancy reduction hypothesis does in fact also minimize the discrete entropy of a uniformly quantized code. In particular, if we look at the discrete entropy of the four different transforms, oPCA, wPCA, wICA, nICA (Figure 7B), we find that asymptotically the two PCA transforms require slightly more entropy than the two ICA transforms, and there is no difference anymore between oPCA and wPCA or wICA and nICA. This close relationship between discrete and differential entropy for high-rate quantization, however, is not sufficient to determine the coding performance evaluated by the rate–distortion curve. The latter requires to compare also the reconstruction error for the given quantization.

#### Reconstruction error

The reconstruction error is defined as the mean squared distance in the pixel basis between the original image and the image obtained by reconstruction from the quantized coefficients of the considered transformation. For the reconstruction, we simply use the inverse of the considered transformation, which is optimal in the limit of high-rate quantization.

**Figure 8:**
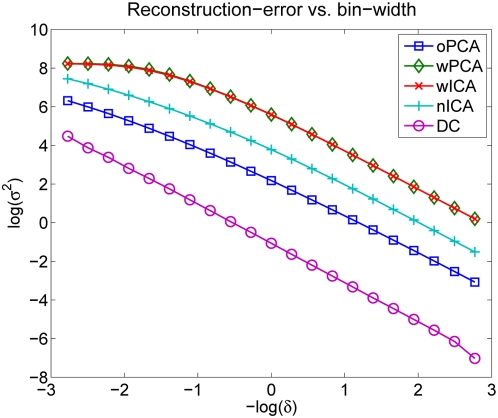
Reconstruction Error vs. Bin Width of Discrete Entropy. Reconstruction error 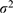 as a function of the bin width 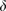, shown on a logarithmic scale. The differences between the different transforms are relatively large. Only the two transformations with exactly the same metric, wPCA and wICA, exhibit no difference in the reconstruction error.

When looking at the reconstruction error as a function of the bin width (Figure 8) we can observe much more pronounced differences between the different transformations than it was the case for the entropy. As a consequence, the differences in the reconstruction error turn out to be much more important for the rate-distortion trade-off than the differences in the entropy. Only the two transformations with exactly the same metric, wPCA and wICA, exhibit no difference in the reconstruction error. This suggests that minimization of the multi-information is strictly related to efficient coding if and only if the transformation with respect to the pixel basis is orthogonal. As we have seen that the potential effect of higher-order redundancy reduction is rather small, we expect that the PCA transform constitutes a close approximation to the minimizer of the multi-information among all orthogonal transforms because PCA is the only orthogonal transform which removes all second-order correlations.

## Discussion

The structural organization of orientation selectivity in the primary visual cortex has been associated with self-organization since the early seventies [55], and much progress has been made to narrow down the range of possible models compatible with the empirical findings (e.g., [56]–[58]). The link to visual information processing, however, still remains elusive [59]–[61].

More abstract unsupervised learning models which obtain orientation selective filters using sparse coding [8] or ICA [23] try to address this link between image processing and the self-organization of neural structure. In particular, these models not only seek to reproduce the orientation tuning properties of V1 simple cells but they additionally address the question of how the simple cell responses collectively can instantiate a representation for arbitrary images. Furthermore, these image representations are learned from an information theoretic principle assuming that the learned filters exhibit advantageous coding properties.

The goal of this study is to quantitatively test this assumption in the simple linear transform coding framework. To this end, we investigated three criteria, the multi-information—i.e., the objective function of ICA—the average log-loss, and rate-distortion curves. There are a number of previous studies which also aimed at quantifying how large the advantage of the orientation selective ICA filters is relative to second-order decorrelation transformations. In particular, four papers [24]–[26],[28], are most closely related to this study as all of them compare the average log-loss of different transformations. However, they did not provide a coherent answer to the question how large the advantage of ICA is compared to other decorrelation transforms.

Lewicki and Olshausen [24] found that their learned bases show a 15–20% improvement over traditional bases. However, their result cannot be used to compare second-order and higher-order redundancy reduction because the entire analysis is based on a dataset in which all images have been preprocessed with a bandpass filter as in olshausen:1996. Since bandpass filtering already removes a substantial fraction of second-order correlations in natural images, their study is likely to systematically underestimate the total amount of second-order correlations in natural images.

Lee et al. [25],[26] reported an advantage of over 100% percent for ICA in the case of color images and a more moderate but substantial gain of about 20% for gray-value images. In order to avoid possible differences due to the choice of data set we here used exactly the same data as in [25],[26]. Very consistently, we find only a small advantage for ICA of less than five percent for both multi-information and the average log-loss. In particular, we are not able to reproduce the very large difference between color and gray-value images that they reported. Unfortunately, we cannot pinpoint where the differences in the numbers ultimately come from because it is not clear which estimation procedure was used in [25],[26].

The estimators used for the measurements in the present study have been shown previously to give correct results on artificial data [28] and we provide our code online for verification. Furthermore, Weiss and Freeman showed for an undirected probabilistic image model that whitening already yields 98% of the total performance [62]. Finally, the superior performance of the simple SSD model with only two free parameters provides a very strong explanation for why the gain achieved with ICA is so small relative to a random decorrelation transform: Since a spherically symmetric model is invariant under orthogonal transformations and provides a better fit to the data, the actual shape of the filter does not seem to be critical. It also shows that the fundamental assumption underlying ICA—the data are well described by a linear generative model with independent sources—is not justified in the case of natural images.

From all these results, we can safely conclude that the actual gain of ICA compared to PCA is smaller than 5% for both gray level images and color images.

### Is Smaller Than 5% Really Small?

A valid question to ask is whether comparing the amount of higher-order correlations to the amount of second-order correlations is the right thing to do. Even if the amount of higher-order correlations may be small in comparison to the amount of second-order correlations, we still know that higher-order correlations can be a critical signature of the content of an image. For example, textures are very useful to demonstrate how changes in higher-order correlations can change the perceptual meaning of an image.

**Figure 9:**
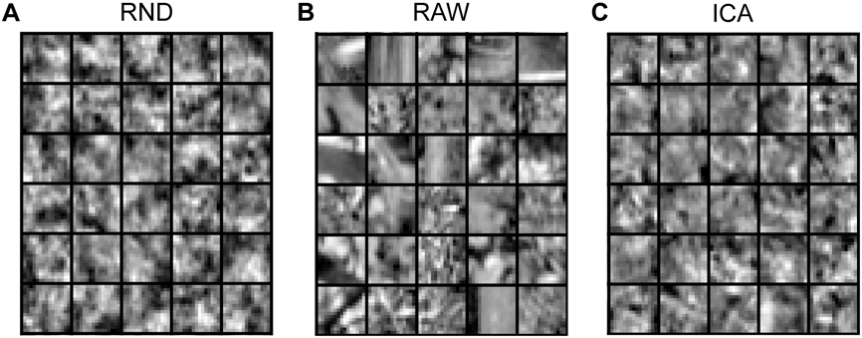
Comparison of Patches Sampled From Different Image Models. The figure demonstrates that the perceptual similarity between samples from the ICA image model (C) and samples from natural images (B) is not significantly increased relative to the perceptual similarity between samples from the RND image model (A) and (B).

Our results on the rate-distortion trade-off can be taken as an indication that the fraction of higher-order correlations captured by ICA is perceptually less relevant. This interpretation is further corroborated by a psychophysical comparison of the *perceptual* redundancy of the ICA and the PCA basis [63]. Another confirmation of this interpretation can be obtained if we use the learned image representations as generative models. Perceptually image patches sampled from the ICA model do not look more similar to natural image patches than those sampled from the random decorrelation basis (Figure 9). Currently, we are running psychophysical experiments which also show quantitatively that there is no significant difference between the ICA model and the PCA model if the subjects have to discriminate between textures that are generated by these models.

In summary, we were not able thus far to come up with a meaningful interpretation for which the improvement of ICA would be recognized as being large. On the basis of the present study it seems rather unlikely that such a measure can be found for linear ICA. Instead, we believe that more sophisticated, nonlinear image models are necessary to demonstrate a clear advantage of orientation selectivity.

### What about Nonparametric Approaches?

The focus on linear redundancy reduction models in this study is motivated by the goal to first establish a solid and reproducible result for the simplest possible case before moving on to more involved nonlinear transformations. Nevertheless, it is important to discuss what we can expect if the restriction to linear transformations is dropped. From a nonparametric analysis [27], Petrov and Zhaoping concluded that higher-order correlations in general contribute only very little to the redundancy in natural images and, hence, are probably not the main cause for the receptive field properties in V1. The empirical support for this claim, however, is limited by the fact that their comparison is based on mutual information estimates within a very small neighborhood of five pixels only. This is problematic as it is known that many kinds of higher-order correlations in natural images become apparent only in much higher-dimensional statistics [64]. Furthermore, their estimate of the amount of second-order correlations is not invariant against pointwise nonlinear transformations of the pixel intensities.

In a more recent non-parametric study, Chandler and Field arrived at a very different result regarding the relative contribution of second-order and higher-order dependencies [29]. They use nearest-neighbor based methods to estimate the joint entropy of natural images in comparison to “spectrum-equalized” noise and white noise, where “spectrum-equalized” noise denotes Gaussian noise with exactly the same spectrum as that of natural images. As shown in Figure 18 of [29] they find a smaller difference between spectrum-equalized noise and white noise than between natural images and spectrum-equalized noise. Hence, from their finding, it seems that the amount of higher-order correlations in natural images is even larger than the amount of second-order correlations. Also this result has to be taken with care: Reliable non-parametric estimates in high-dimensions are difficult to obtain even if one resorts to nearest-neighbor based methods, and the estimate of the amount of second-order correlations in [29] is not invariant against pointwise nonlinear transformations of the pixel intensities, too.

In summary, the present nonparametric studies do not give a unique answer regarding the total amount of higher-order correlations in natural images. Since estimating the absolute amount of multi-information is an extremely difficult task in high dimensions, the differences in the results can easily originate from the different assumptions and approximations made in these studies. Consequently, it remains an open question how large the true total redundancy of natural images is. In any case, it is clear that there are many higher-order redundancies in natural images that play a crucial role for visual perception. No matter how large these redundancies are in comparison to the second-order correlations, we need to develop better image models that have the right structure to capture these regularities.

### What about Nonlinear Image Models?

Apart from the non-parametric approaches, a large number of nonlinear image models has been proposed over the years which are capable to capture significantly more statistical regularities of natural images than linear ICA can do (e.g., [62], [65]–[72]). In fact, Olshausen and Field [8] already used a more general model than linear ICA when they originally derived the orientation selective filters from higher-order redundancy reduction. In contrast to plain ICA, they used an *overcomplete* generative model which assumes more source signals than pixel dimensions. In addition, the sources are modeled as latent variables like in a factor analysis model. That is the data is assumed to be generated according to 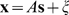 where 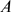 denotes the overcomplete dictionary, 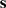 is distributed according to a sparse factorial distribution, and 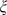 is a Gaussian random variable. The early quantitative study by Lewicki and Olshausen [24] could not demonstrate an advantage of overcomplete coding in terms of the rate-distortion trade-off and also the more recent work by Seeger [70] seems to confirm this conclusion. The addition of a Gaussian random variable 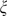 to 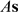, however is likely to be advantageous as it may help to interpolate betweem the plain ICA model on the one hand and the spherically symmetric model on the other hand. A comparison of the average log-loss between this model and plain ICA has not been done yet but we can expect that this model can achieve a similar or even better match to the natural image statistics as the spherically symmetric model.

The spherical symmetric model can also be modeled by a redundancy reduction transformation which changes the radial component such that the output distribution is sought to match a Gaussian distribution [31]. Hence, the redundancy reduction of this model is very similar to the average log-loss of the spherically symmetric distribution. From a biological vision point of view, this type of model is particularly interesting as it allows one to draw a link to divisive normalization, a prominent contrast gain control mechanism observed for virtually all neurons in the early visual system. Our own ongoing work [30] shows that this idea can be generalized to a larger class of 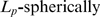 symmetric distributions [67]. In this way, it is possible to find an optimal interpolation between ICA and the spherically symmetric case [73]. That is, one can combine orientation selectivity with divisive normalization in a joint model. Our preliminary results suggests that optimal divisive normalization together with orientation selectivity allows for about 10% improvement while divisive normalization alone (i.e., the spherical symmetric model) is only 2% worse [30].

### Concluding Remarks

Taken together, the effect of orientation selectivity on redundancy reduction is very limited within the common linear filter bank model of V1 simple cells. In contrast to Zhaoping and coworkers, we do not claim that higher-order redundancy minimization is unlikely to be the main constraint in shaping the cortical receptive fields [22],[27]. Our conclusion is that although there are significant higher-order correlations in natural images, orientation selective filtering turns out to be not very effective for capturing these. Nevertheless, we do expect that visual representations in the brain aim to model those higher-order correlations, because they are perceptually relevant. Therefore, we think it is important to further explore which type of nonlinear transformations would be suitable to capture more pronounced higher-order correlations. The objective functions studied in this paper are related to factorial coding, density estimation and minimization of the pixel mean square reconstruction error. Of course, there are also other alternatives that are interesting, too. For example, Zhaoping proposed that one possible goal of V1 is to explicitly represent bottom-up saliency in its neural responses for visual attentional selection [12]. As a further alternative, we are currently trying to extend the efficient coding framework to deal with other loss functions. Obviously, the goal of the visual system is not to preserve the pixel representation of the visual input. Instead, seeing serves the purpose to make successful predictions about behaviorally relevant aspects of the environment [74]. Since 3D shape inference is necessary to almost any naturally relevant task, it seems particularly interesting to explore the role of orientation selectivity in the context of 3D shape inference [75]. For a quantitative account of this problem one can seek to minimize the reconstruction error for the 3D shape rather than for its 2D image. Certainly, this task is much more involved than image reconstruction. Nevertheless, we need to think more about how to tackle the problem of visual inference within the framework of unsupervised learning in order to unravel the principles of neural processing in the brain that are ultimately responsible for our ability to see.

## Supporting Information

Text S1 In the article we chose a patch size of 7×7 in order to enhance the comparability to previous work. The supplementary material contains all results (figures and tables) for patch size 15×15.
(2.82 MB PDF)
