# Supplementary material for: Natural Image Coding in V1: How Much Use Is Orientation Selectivity?
Source: PLoS Comput Biol. 2009 Apr 3;5(4):e1000336. doi: 10.1371/journal.pcbi.1000336 (PMC2658886; doi:10.1371/journal.pcbi.1000336)
Supplement: Text S1 — In the article we chose a patch size of 7×7 in order to enhance the comparability to previous work. The supplementary material contains all results (figures and tables) for patch size 15×15. (2.82 MB PDF) [file pcbi.1000336.s001.pdf]

Additional Material

August 14, 2008

Figures and Tables for Patch Size 15×15

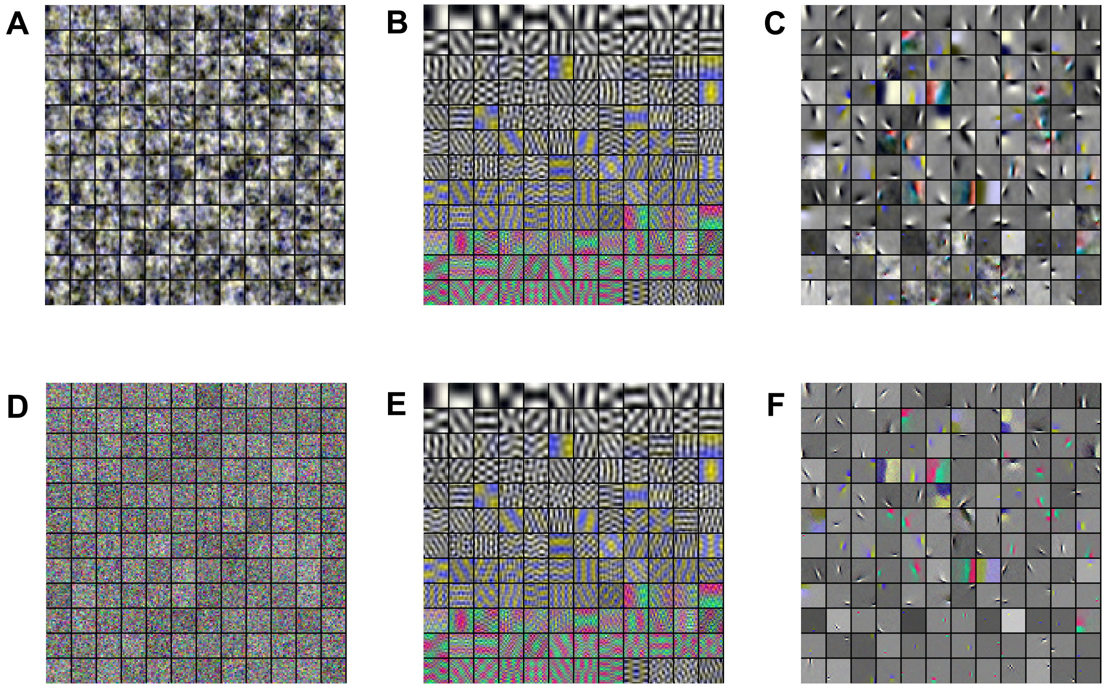

Figure 1: **Examples for Receptive Fields of Various Image Transforms** Basis functions of a random decorrelation transform (**RND**), principal component analysis (**PCA**) and independent component analysis (**ICA**) in pixel space (**A**-**C**) and whitened space (**E**-**F**). The image representation in whitened space is obtained by left multiplication with the matrix square root of the inverse covariance matrix r$C^{-1/2}$. This figure can only give a rough idea of the shape of the basis functions. For a detailed inspection of the basis functions we refer the reader to our web page `http://www.kyb.mpg.de/bethge/code/QICA/` where we provide all the data and code used in this paper.

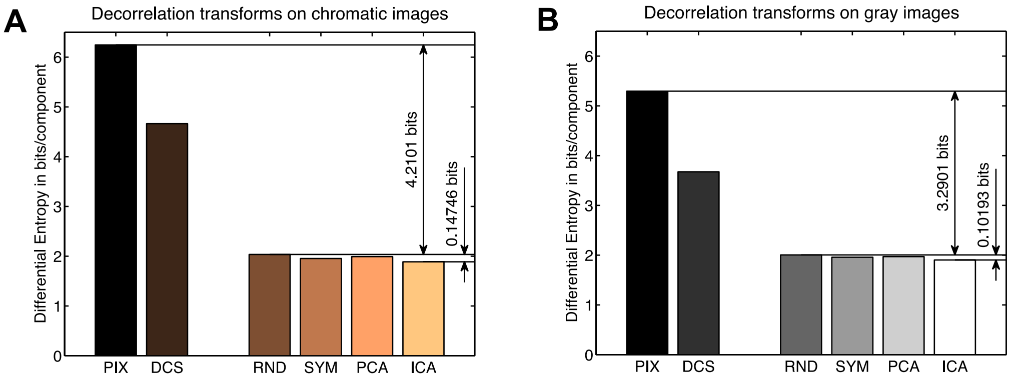

Figure 2: **Multi-Information Reduction per Dimension** Average differential entropy $\langle h \rangle$ for the pixel basis (PIX), after separation of the DC component (DCS), and after application of the different decorrelation transforms. The diagram shows the results for chromatic images (**A**) and the diagram for gray value images (**B**). For both types of images, only a marginal amount can be accounted to the reduction of higher order dependencies.

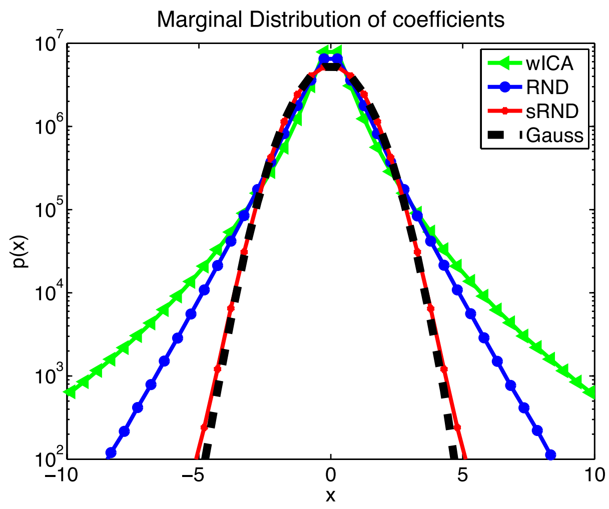

Figure 3: **The Distribution of Natural Images does not Conform with the Generative Model of ICA** In order to test for statistical dependencies among the coefficients Y_{wICA} of whithened ICA for single data samples, the coefficients were shuffled among the data points along each dimension. Subsequently, we transform the resulting data matrix Y_{sICA} into $Y_{\mathrm{sRND}} = W_{RND} W_{wICA}^{-1} Y_{\mathrm{sICA}}$. This corresponds to a change of basis from the ICA to the random decorrelation basis (RND). The plot shows the log-histogram over the coefficients over all dimensions. If the assumptions underlying ICA were correct, there would be no difference between the histogram of Y_{sRND} and Y_{RND}.

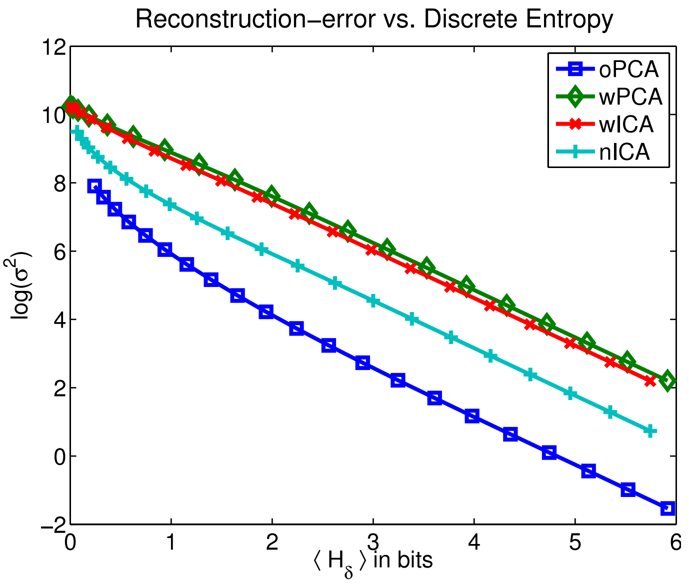

Figure 4: **Rate-distortion Curves** Rate-distortion curve for PCA and ICA when equalizing the output variances (wPCA and wICA) and when equalizing the norm of the corresponding image bases in pixel space (oPCA and nICA). The plot shows the discrete entropy H_δ in bits (averaged over all dimensions) against the log of the squared reconstruction error σ^2. oPCA outperforms all other transforms in terms of coding efficiency. wPCA in turn performed the worst and remarkably similar to wICA. Since wPCA and wICA differ only by an orthogonal transformation, both representations are bound to the same metric. oPCA is the only transformation which has the same metric as the pixel representation according to which the reconstruction error is determined. By normalizing the length of the ICA basis vectors in the pixel space, the metric of nICA becomes more similar to the pixel basis and the performance with respect to coding efficiency can be seen to improved considerably.

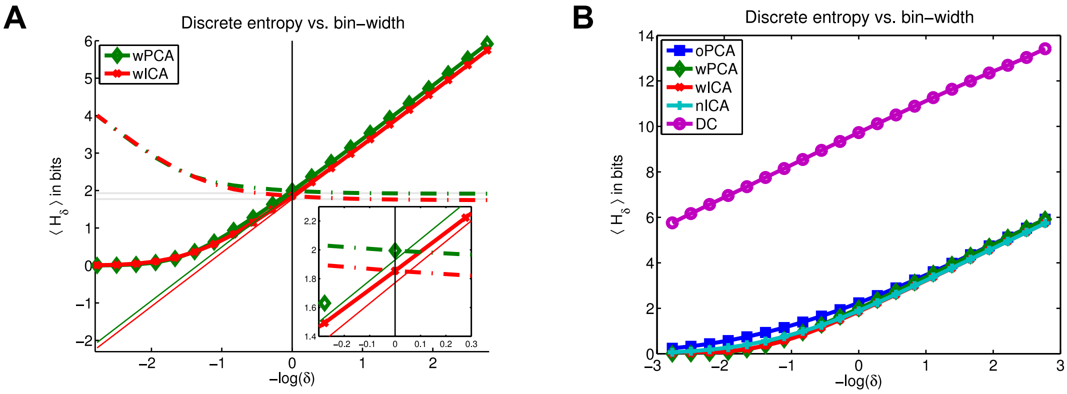

Figure 5: **Discrete vs. Differential Entropy** **A.** Relationship between discrete and differential entropy. Discrete entropy $\langle H_\delta \rangle$ averaged over all channels as a function of the negative log-bin-width. The straight lines constitute the linear approximation to the asymptotic branch of the function. Their interception with the y-axis are visualized by the gray shaded, horizontal lines. The dashed lines represent $\langle h_\delta \rangle$ which converge to the gray shaded lines for $\delta \rightarrow 0$. **B.** There are only small differences in the average discrete entropy for oPCA, wPCA, wICA, nICA as a function of the negative log-bin-width. Since the discrete entropy of the DC component is the same for all transforms, it is not included in that average but plotted separately instead.

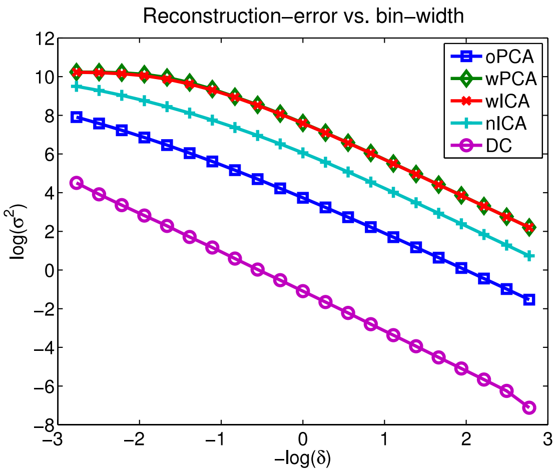

Figure 6: **Reconstruction Error vs. Bin Width of Discrete Entropy** Reconstruction error σ^2 as a function of the bin-width δ, shown on a logarithmic scale. The differences between the different transforms are relatively large. Only the two transformations with exactly the same metric, wPCA and wICA, exhibit no difference in the reconstruction error.

**Absolute Difference**			**Relative Difference**		
	Color	Gray		Color	Gray
RND-PIX	-4.2101 ± 0.0020	-3.2901 ± 0.0019			
SYM-RND	-4.2915 ± 0.0023	-3.3360 ± 0.0022	$\frac{\text{SYM-RND}}{\text{SYM-PIX}}$	1.90 ± 0.01	1.37 ± 0.01
PCA-RND	-4.2534 ± 0.0022	-3.3239 ± 0.0022	$\frac{\text{PCA-RND}}{\text{PCA-PIX}}$	1.02 ± 0.01	1.01 ± 0.01
ICA-RND	-4.3575 ± 0.0024	-3.3921 ± 0.0026	$\frac{\text{ICA-RND}}{\text{ICA-PIX}}$	3.38 ± 0.02	3.01 ± 0.02

Table 1: **Comparision of the Multi-Information Reduction for Chromatic and Achromatic Images** Differences in the multi-information reduction between various decorrelation transforms (SYM, PCA, ICA) relative to a random decorrelation transform (RND) compared to the multi-information reduction achieved with the random decorrelation transform relative to the original pixel basis (RND-PIX). The *absolute* multi-information reduction is given in bits/component on the left hand side. How much more the special decorrelation transforms SYM, PCA and ICA can reduce the multi-information *relative* to the random (RND) one is given in percent on the right hand side.

	**Color**		**Gray**	
**A**	ALL		ALL	
RND	1.9925 ± 0.0041		1.9685 ± 0.0038	
SYM-RND	-0.1203 ± 0.0007		-0.0682 ± 0.0005	
PCA-RND	-0.0511 ± 0.0004		-0.0364 ± 0.0005	
ICA-RND	-0.1829 ± 0.0009		-0.1191 ± 0.0010	
SSD-RND	-0.2461 ± 0.0022		-0.2742 ± 0.0030	
**B**	DLL	$\langle\alpha\rangle$	DLL	$\langle\alpha\rangle$
RND	-0.0086 ± 0.0002	1.1273 ± 0.0039	-0.0060 ± 0.0004	1.0811 ± 0.0034
SYM	-0.0472 ± 0.0005	0.9034 ± 0.0027	-0.0282 ± 0.0006	0.9535 ± 0.0032
PCA	-0.0162 ± 0.0003	1.0229 ± 0.0033	-0.0085 ± 0.0005	1.0100 ± 0.0031
ICA	-0.0434 ± 0.0004	0.7540 ± 0.0019	-0.0227 ± 0.0007	0.8237 ± 0.0025

Table 2: **Comparision of the Average Log-Loss (ALL) and the Differential Log-Likelihood (DLL) Chromatic and Achromatic Images** **A.** The first row shows the average log-loss (ALL, in bits/component) of the density model determined by the linear transformation RND. The value was obtained by averaging over 10 separately sampled training and test sets of size 40.000 and 50.000, respectively. The following rows shows the difference of the ALL of the models SYM, PCA, ICA and the spherically symmetric density (SSD) to the ALL determined by linear transformation RND. The large value for RND−ICA fundamentally contradicts the assumptions underlying the ICA model. **B.** The small DLL values suggest, that the exponential power distribution fits the shape of the individual coefficient distributions well. In addition, we also report the average exponent $\langle\alpha\rangle$ of the exponential power family fit to the individual coefficient distributions ($\alpha = 1$ corresponds to a Laplacian shape).

Multi Information for Small Patches

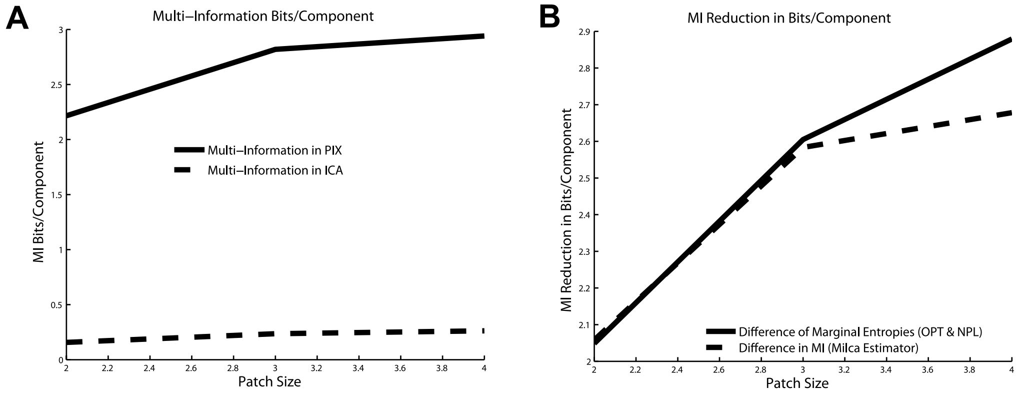

Figure 7: **Multi-Information for Small Patches** **A** Multi-information for patch sizes 2×2, 3×3 and 4×4 in the representations PIX and ICA. **B** Multi-information reduction as estimated by the multi-information from the left plot and by the differences in the marginal entropies. For patch size 4×4, the estimations start to disagree. Since the multi-information is much harder to estimate than the marginal entropies, we conclude that from patch size 4×4 on, the multi-information estimates are not reliable anymore.

	2×2	3×3	4×4
PIX	2.2157	2.8193	2.9405
ICA	0.1573	0.2358	0.2622

Table 3: **Multi-Information for Small Patch Sizes** The table shows the multi-information in the representations PIX and ICA in bits/pixel as computed with the estimator from the MILCA package by Kraskov.
